# Reactive Carbonyl Species Scavenger: Epigallocatechin-3-Gallate

**DOI:** 10.3390/foods13070992

**Published:** 2024-03-25

**Authors:** Haiying Luo, Juanying Ou, Junqing Huang

**Affiliations:** 1 Department of Food Science and Engineering, Jinan University, Guangzhou 510632, China; haiyinglo@163.com (H.L.); toujy@jnu.edu.cn (J.O.); 2 School of Traditional Chinese Medicine, Jinan University, Guangzhou 510632, China

**Keywords:** epigallocatechin-3-gallate, reactive carbonyl species, adduct, polyphenols

## Abstract

Epigallocatechin-3-gallate (EGCG), a prominent polyphenol found abundantly in tea, has garnered significant attention for its potential in preventing and ameliorating a wide range of diseases. Its remarkable antioxidant properties and ability to capture reactive carbonyl species make it a key player among tea’s polyphenolic components. This paper delves into the synthesis and origins of both EGCG and reactive carbonyl species (RCS), emphasizing the toxicity of RCS in various food sources and their formation during food processing. Understanding EGCG’s capability to capture and metabolize RCS is crucial for harnessing its health benefits. Thus, this paper explores the underlying mechanisms of EGCG for RCS inhibition and its role in capturing these compounds to generate EGCG-RCS adducts. And the absorption and metabolism of EGCG-RCS adducts is also discussed.

## 1. Introduction

The health benefits of tea, derived from Camellia sinensis sinensis and Camellia sinensis assamica, have been extensively recognized, with variations like green tea, black tea, white tea, and oolong tea based on fermentation levels [1]. Tea has gained recognition for its multifaceted health benefits, including cancer treatment [2], cardiovascular protection [3], antiviral [4], anti-inflammatory [5], immunomodulatory effects [6] and prevention of central degenerative diseases [7]. The composition of active compounds in teas varies upon fermentation levels. These advantages are attributed to the presence of tea polyphenols [8], including catechins, caffeine, and theanine, with EGCG standing out for its highest activity among catechins. EGCG has a chemical structure featuring one pyran C ring, 5,7-dihydroxy-substituted A ring, and trihydroxyphenol structures on the B and D rings. With eight phenolic hydroxyl structures, EGCG demonstrates potent activity, chelating small molecules and aiding in their elimination.

RCS encompass one or more carbonyl structures, including α,β-unsaturated aldehydes, dialdehydes, ketoaldehydes, and notable examples such as formaldehyde (FA), glyoxal (GO), methylglyoxal (MGO), malondialdehyde (MDA), acrolein (ACR), and 4-hydroxy-2-nonenal (HNE). Currently, almost thirty types of RCS are recognized, originating from a diverse array of sources categorized as endogenous and exogenous [9]. Exogenous RCS sources are primarily divided into food-derived and environmental origins. During the storage and fermentation of common foods like bread, milk, and coffee, RCS generation is frequent. Environmental RCS sources encompass exhaust emissions, and industrial pollution, among others. These external RCS can enter organisms through inhalation, contact, ingestion, or drinking, subsequently altering an organism’s RCS concentration. Endogenous RCS largely arise as by-products of enzymatic or peroxidation reactions involving sugars, lipids, and certain proteins. They are closely intertwined with non-glycosylation reactions and oxidative stress within the body [10]. In general, exogenous dietary intake is the main source of ingested RCS [11]. Once the human body is in a sub-healthy state, such as diabetes and dyslipidemia, elevated circulating levels of sugar and fat metabolism lead to RCS production and accumulation [12].

Characterized by strong electrophilic properties, RCS attracts nucleophiles like hydroxyls and imidazoles. On one hand, RCS offers merits such as enhancing flavor in food and displaying antimicrobial activity [13]. On the other hand, the perilous nature of RCS prevails, often being viewed as a precursor to advanced end-stage products of late-glycosyl metabolism. This exposure brings about risks, including mitochondrial toxicity, cytotoxicity, genotoxicity, and carcinogenicity. Associations have been established with conditions like non-alcoholic fatty liver disease, end-stage liver cancer, atherosclerosis [14], diabetes mellitus [15], Alzheimer’s disease [16], and chronic obstructive pulmonary disease [17].

Multiple strategies are employed to counteract the detrimental impacts of RCS. Common approaches include the utilization of natural eliminators like dietary polyphenols, protein binding to mitigate toxicity, and the regulation of signaling pathways to enhance detoxification enzyme activity. The swift and efficient elimination of RCS forms a significant realm of research. This paper focuses on highlighting the role of EGCG in responding to unsaturated aldehydes, detailing the EGCG-driven RCS elimination process, and shedding light on the subsequent metabolic pathway. These insights contribute to our comprehension of how to alleviate complications associated with RCS.

## 2. RCS in Food Processing

### 2.1. Species and Toxicity

The majority of RCS are generated during food processing, with only a limited number of natural foods naturally containing RCS, including cucumber, guava, and fresh pistachios. Typically, volatile compounds in natural foods encompass RCS, originating from plants through a series of physiological and biochemical reactions. Common α,β-unsaturated carbonyl compounds found in food include ACR, 4-hydroxynonenal (HNE), MDA, and 4-hydroxy-2-hexenal (HHE), among others. Interestingly, the combined consumption of various foods, such as smoking and alcohol consumption, can amplify the concentration of RCS in the bloodstream, showcasing a synergistic effect [18]. 

**Figure 1:**
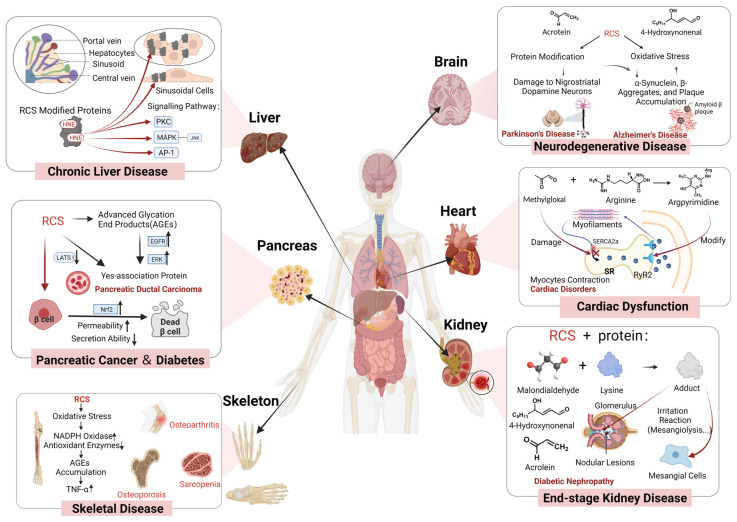
RCS and the by-products of RCS are highly reactive with cellular components and exhibit toxicity. The toxicity involves multiple systems and organs (↑ indicates an increase in a certain indicator; ↓ indicates a decrease in a certain indicator).

RCS has the ability to alter the structure and functionality of biomolecules, including amino acids, nucleic acids, and vitamins. This modification results in the compromise of their biological functions, leading to toxicity. Moreover, RCS can instigate oxidative stress, inflammation, and apoptosis in cells, thereby impairing physiological functions [11,19] (Figure 1). For instance, RCS-induced modifications in proteins lead to receptor dysfunction, affecting calcium ion signaling. Increased RyR2 phosphorylation caused by carbonyl/oxidative stress may contribute to heart failure, arrhythmias, and diabetic complications [20]. Elevated levels of HNE can affect the activities of proteins like protein kinase C (PKC) and mitogen-activated protein kinase (MAPK) [20]. ACR exerts modifications on a multitude of functional proteins, including redox-regulated proteins, cytoskeletal proteins, transcription factors, and other critical proteins involved in signaling [21]. It has been suggested that ACR might impede endogenous antioxidant defenses by directly depleting glutathione, inhibiting the thioredoxin system, or suppressing antioxidant enzyme activity. Furthermore, ACR facilitates the assembly of the cytoplasmic subunit of nicotinamide adenine dinucleotide phosphate (NADPH) oxidase into membranes, thereby activating NADPH enzymes and inciting oxidative stress in vivo, which ultimately leads to oxidative damage [22]. Research has proposed that ACR disrupts molecular regulation of mitochondrial metabolism in rat lungs in a dose-dependent manner [23]. This disruption involves alterations in the transcript abundance of two subunits within the electron transport chain complex and regulatory factors linked to mitochondrial biogenesis, consequently leading to mitochondrial toxicity. Moreover, approximately 30% of HNE-modified proteins are associated with mitochondria. Following HNE modification, there is a reduction in mitochondrial fusion proteins, like optic atrophy-1 (OPA1) and mitofusin-2 (MFN2), while there is an increase in protein levels of fission proteins, particularly p-Dynamin-1-like (DNM1L) and DNM1L [24]. This shift contributes to mitochondrial morphology changes characterized by shortened structures, ultimately compromising the normal bioenergetic efficiency of the mitochondria.

### 2.2. Protein Oxidation

Protein carbonylation is an irreversible process, which typically occurs as a consequence of oxidative stress within a pro-oxidative environment. This modification introduces carbonyl derivatives, including aldehydes and ketones, into proteins, primarily through the direct oxidation of specific amino acid side-chains. In foods, the RCS can be generated via two mechanisms in the process of protein oxidation, including oxidative deamination of amino acids by a radical-mediated direct mechanism, known as the ‘Stadtman’ pathway, and by oxidative cleavage of the peptide backbone via the α-amidation pathway or via oxidation of glutamyl side chains [25]. It is generally assumed that the latter among these two mechanisms is of negligible importance. Notably, in the ‘Stadtman’ pathway, under the direct influence of reactive oxygen species (ROS), alkaline amino acids including proline (Pro), arginine (Arg), lysine (Lys), and threonine (Thr) can be profoundly affected, leading to the formation of various carbonylated compounds. The ROS initiate their attack on the ε-amino moiety of alkaline amino acids by abstracting a hydrogen atom from the neighboring carbon. This process results in the initial formation of an imino group, which is an intermediate and unstable product. Subsequently, this intermediate product readily undergoes hydrolysis to form the corresponding protein carbonyl. According to this reaction pathway, proline can yield 2-pyrrolidone, while the combination of arginine and proline can produce glutamic semialdehyde. Additionally, lysine generates α-aminoadipic semialdehyde, and threonine leads to the formation of 2-amino-3-ketobutyric acid [26]. 

Mammals undergo endogenous oxidative metabolism to produce unsaturated carbonyl compounds such as GO, MGO, Glycolaldehyde, ACR, 2-Hydroxypropanal, which are mainly derived from the oxidative catabolism of threonine and glycine, and the metabolism of polyamine catabolism. Specifically, Semicarbazide-sensitive amine oxidase (SSAO), also known as vascular adhesion protein 1 (VAP-1), which is highly expressed in blood vessels and adipocytes, is the enzyme that catalyzes the generation of unsaturated carbonyl compounds. The metabolite of glycine and threonine is aminoacetone, and SSAO can catalyze the deamination reaction using aminoacetone as a substrate to produce hydrogen peroxide and ammonia along with formaldehyde and methylglyoxal, respectively [27]. In addition, myeloperoxidase secreted by activated phagocytes catalyzes the oxidation of L-threonine to 2-hydroxypropanal, which undergoes a reaction of dehydrogenation of carbon dioxide and water to produce acrolein [28]. As myeloperoxidase is released in phagocytes, acrolein tends to accumulate at sites of inflammation. Given that the body lacks the ability to synthesize threonine, it relies on dietary or alternative sources to fulfill its protein requirements. Foods of animal origin are rich in threonine and glycine [29]. Interestingly, the intake of threonine exhibits only a weak positive correlation with blood concentrations of RCS, while there is a negative correlation between glycine intake and blood concentrations of RCS [30].

Polyamines are generally found in mammals as spermidine, spermine, and their precursor putrescine, usually in the RNA-bound form, and are released from ribosomes when cells are damaged. Spermine oxidase and acetylpolyamine oxidase are two enzymes that oxidize polyamines to generate ACRs, the former using spermine as a substrate while the latter preferring N1-acetylspermidine and N1-acetylspermine as substrates. Specifically, the flavoprotein spermine oxidase (SMO) catalyzes the oxidation of spermine to spermidine and 3-aminopropanal, 3-aminopropanal [31,32]. The other is the spermidine/spermine N1-acetyltransferase (SSAT), adding an acetyl group to the aminopropyl terminus of spermine and spermine, making it a substrate for acetylpolyamine oxidase (APAO), which forms N-acetyl-3-aminopropanal and smaller polyamines, and further 3-aminopropanal. ACR produces 3-aminopropanal primarily by a non-enzymatic reaction [33].

### 2.3. Lipid Oxidation

The critical aspects of lipid peroxidation in food processing are associated with exposure to elevated temperatures, leading to the accelerated cleavage of polyunsaturated fatty acids within cholesterol esters, phospholipids, and triglycerides. This process generates numerous smaller fragments, ultimately resulting in the production of significant quantities of RCS [34]. When oxygen attacks the unsaturated fatty acids in cell membranes or lipoproteins, the free radicals are produced. These free radicals further react with other molecules in foods, forming a series of lipid peroxides (LOOH). LOOH are decomposed into dialdehydes (like malondialdehyde MDA, ketoaldehydes, α,β-unsaturated aldehydes) and enals (4-hydroxynonenal HNE, acrolein), under the catalysis of metal ions or enzymes [19]. Among these, RCS, MDA, and HNE are commonly found in fatty foods. HNE, generated from the peroxidation of omega-6 polyunsaturated fatty acids like linoleic acid (LA), gamma-linolenic acid (GLA), and arachidonic acid (AA), and MDA, formed through the oxidation of unsaturated fatty acids such as linoleic acid, linolenic acid, and arachidonic acid, are prevalent in various food items [35]. Thus, the rate and extent of lipid oxidation and RCS formation in foods depend on many factors, such as the type and amount of lipids, antioxidants, oxygen, light, heat, metal ions, pH, water activity, and food structure. Food processing can also affect lipid oxidation and RCS formation, either positively or negatively. For instance, heating can speed up these reactions, but it can also kill enzymes and microorganisms that can catalyze them. At low temperatures, lipid peroxidation is also capable of generating RCS. A recent study indicated that the number of freeze-thaw cycles meat undergoes correlates with increased fat oxidation in raw meat and heightened RCS production [36]. This phenomenon could be attributed to the interplay of the Maillard reaction and lipid oxidation occurring at lower temperatures.

### 2.4. Maillard Reaction

**Figure 2:**
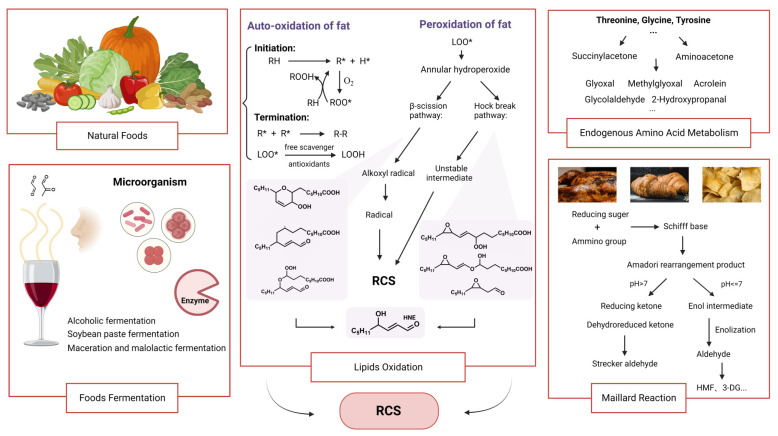
The source of reactive carbonyl species (RCS) from foods mainly including: natural foods without food processing, fermentation, lipids oxidation, Maillard reaction, and endogenous amino acid metabolism.

The Maillard reaction is usually divided into three stages. During the early stage of the Maillard reaction, nucleophilic addition of the carbonyl group on a reducing sugar generates N-substituted glycosylamine, which subsequently undergoes rearrangement to form Amadori compounds. In the intermediate stage, under pH conditions greater than 7, rearrangement products yield reducing ketones and dehydroreduced ketone, while Strecker aldehyde forms through Strecker degradation. Alternatively, when the pH is below 7, intermediate enols arise from Amadori rearrangement products, which further degrade into aldehydes like furan aldehydes and methylfuran aldehydes. This process leads to the formation of compounds such as 5-Hydroxymethylfurfural (HMF), 1-deoxyglucosone (1-DG), 3-deoxyglucosone (3-DG), and reductones through enolization. At this stage, both acidic and basic conditions are likely to produce more unsaturated carbonyl compounds. The active product’s carbonyl group can react with free amino groups to form Strecker degradation products, a pivotal step in generating food flavors. In the terminal stage, a subsequent series of reactions yield alpha, beta-unsaturated aldehydes or ketones, as well as melanin [37]. Different combinations of reducing sugars with amino acids are one of the important factors affecting the production of RCS, such as two-by-two combinations of glucose, fructose, maltose, lactose and lysine, threonine, cysteine, and serine [38]. It was demonstrated that the highest concentration of unsaturated carbonyl compounds was produced in the fructose–threonine reaction model and the lowest in the lactose–cysteine reaction model. In the glucose–leucine model, 1-DG and 3-DG are formed from enol intermediates from the intermediate stage of the Maillard reaction, 1-DG fission to glycolaldehyde followed by oxidation to GO, while 3-DG has the potential to generate HMF [39]. In the lactulose–lysine model, which is common in dairy products, the early stage of the Maillard reaction produces lactulose–lysine, which undergoes, in turn, acid hydrolysis to form a furosine product, which has a keto group attached to the furan. In the same food model, differences in reaction conditions, such as cooking methods, may have an effect on the amount and type of RCS in the products of the meridian reaction [40]. Several reports indicate that cooking methods such as frying, baking, and grilling produce more Maillard products than steaming, which means that there are more unsaturated carbonyl compounds in the food (Figure 2) [41,42].

### 2.5. Fermentation

Besides lipid oxidation and the Maillard reaction in frying, baking, or roasting, fermentation is another process that can affect the RCS content in foods, depending on the type of food, the type of microorganism, and the fermentation conditions. During the fermentation of certain foods like beer and yogurt, RCS such as MGO, alpha-oxoaldehyde, pyruvic aldehyde, and furan aldehyde are generated. Fermentation can reduce the RCS content in foods by breaking down the lipid or sugar precursors or the RCS itself, by making antioxidants that can capture the RCS, or by creating compounds with the RCS that can lower its toxicity [43,44]. However, fermentation can also increase the RCS content in foods by enhancing the lipid or sugar degradation, by making aldehydes that can react with the RCS, or by boosting the Maillard reaction that can produce the RCS [45]. Sometimes, fermentation can have no noticeable effect on the RCS content in foods if the microorganism has low activity or specificity for the RCS or its precursors, or if the fermentation conditions are not suitable for RCS formation or degradation. Therefore, the effects of fermentation on the RCS content in foods are complex and variable, and they need to be evaluated case by case. In the winemaking process, the two main fermentation processes, including alcoholic fermentation carried out by Saccharomyces cerevisiae and malolactic fermentation performed by lactic acid bacteria Leuconostoc oenos, are responsible for the majority of RCS production. Interestingly, the concentration of certain compounds in wine after fermentation differs based on the yeast/lactic acid bacteria combination used. However, the concentration of aldehydes like GO and MGO, as well as volatile sulfur compounds like dimethyl sulfide (DMS) and hydrogen sulfide (H2S), remains constant regardless of the microbial combination [46]. In the malolactic fermentation process, an important enzyme called malolactase features two nicotinamide adenine dinucleotide (NAD)-binding domains and leads to the production of end RCS products, such as acetic acid, diacetyl, acetyl, and pyruvaldehyde [47]. Other fermented foods like Chinese pickled pepper, Northeast soy sauce, and watermelon sauce utilize microbial aminotransferases to convert free amino acids into alpha-ketoacids during fermentation. Various decarboxylases further break down these alpha-ketoacids into corresponding RCS, like some volatile aldehydes, contributing to the distinctive flavors of the food products [48].

## 3. Metabolic Processes and Influencing Factors of RCS Elimination

### 3.1. Endogenous Factors

Endogenous enzymes play a crucial role in metabolizing RCS in humans, thereby attenuating RCS-induced cellular damage and contributing to RCS detoxifications, including aldehyde dehydrogenases (ALDHs), aldo-keto reductases (AKRs), glutathione-S-transferases (GSTs), and cytochrome P450. These enzymes exhibit a wide range of substrate specificities, varying in their metabolic efficiencies and interactions with chemical structures [49]. Higher concentrations of RCS can stimulate the catalytic performance, and the activities of these enzymes are regulated by the food-borne natural compounds. ALDHs are responsible for converting aldehydes into less toxic carboxylic acids for elimination. This includes aldehyde dehydrogenase 1 family member A1 (ALDH1A1) and aldehyde dehydrogenase 1 family member A3 (ALDH1A3), which metabolize active aldehydes into carboxylic acids, and aldehyde dehydrogenase 4 (ALDH4), which can clear reactive aldehydes from both exogenous sources and lipid oxidation [50]. Cytochrome P450 enzymes mainly achieve RCS detoxification by catalyzing the oxidation of a series of aldehydes into the corresponding acids using oxygen and NADPH [51], and the rate of RCS clearing [52] of P450 is the same with aldehyde dehydrogenase and glutathione-S-transferase. AKRs are involved in the reduction of RCS into the corresponding alcohols, which are then excreted from the body [50]. Some studies indicated that AKR7A2 has higher resistance to HNE and moderate resistance to trans, trans-muconaldehyde (MUC) and MGO [53]. In hepatocytes and the heart, GSTs mainly catalyze the conjugation reaction of reduced glutathione (GSH) and RCS to form GSH conjugates [54]. GSTs are activated upon exposure to pro-oxidants and are more active in catalyzing the elimination of RCS. Although conjugates of GSH of RCS are toxic [55], the formation of conjugates of RCS and GSH catalyzed by GSTs is considered a detoxification step. A portion of the conjugate is reduced to GS-DHN by AKR1B1, excreted from the cell [56]. Additionally, GSTs help reduce toxic aldehydes by inhibiting the formation of lipid peroxidation products, aside from direct RCS elimination [50].

In addition to directly eliminating RCS, antioxidant enzymes also reduce superoxide radicals to help slow RCS formation, including glutathione peroxidases (GPx), superoxide dismutase (SOD), and catalase (CAT). The activity of these enzymes is influenced by various factors, such as the type of substrates, interactions with other enzymes, and the tissue’s internal environment. For instance, SOD relies on the synergistic action of CAT and peroxidase to effectively remove free radicals [57]. Superoxide radicals affect the activity of CAT. Reactive oxygen species, superoxide anion, and nitric oxide can inhibit the activity of GPx. GSH as a reaction substrate will affect the working performance of GPx [58]. These interactions and dependencies among antioxidant enzymes and substrates highlight the intricate nature of RCS detoxification.

Carnosine, a histidine-containing dipeptide, has the ability to sequester ACR within the human body [59]. Experiments demonstrated the presence of carnosine–acrolein adducts in volunteers’ urine both before and after carnosine supplementation. The absence of such adducts in the urine of non-supplemented individuals indicated that dietary carnosine facilitates the detoxification of ACR. However, while it is established that carnosine binds to RCS, the exact in vivo binding mechanism remains unclear, potentially involving either direct binding or proteolytic interaction [60]. Despite its demonstrated efficacy, the bioavailability of carnosine remains limited, and plasma concentrations have yet to be quantified.

### 3.2. Exogenous Factors

Phytochemicals in daily foods show the property of trapping RCS, including carnosine, glutathione, and polyphenols. Phenols, including rutin, quercetin, troxerutin, diosmin, diosmetin, hesperidin, and hesperidin, can effectively trap MGO and GO by forming adducts [61]. In the RCS-BSA model in vitro, phenolic compounds exhibited a more significant trapping capacity for MGO compared to GO. From a structure–activity relationship perspective, free hydroxyl groups contribute to the antiglycative effect, the aglycone showed a higher RCS trapping activity than glycosides in both models, such as hesperetin and hesperidin [62]. Furthermore, within the flavonoid class of compounds, specific structural positions have advantages in binding to GO and MGO. For instance, the active group at C-7 in ring A of flavonoids contributes to the antiglycative effect. Meanwhile, methylation of the hydroxyl group at the C-4’ position of the B ring of flavonoids enhances the antiglycative effect. However, the presence of a double bond in ring C decreases the trapping and reducing ability of MGO and GO. It has been reported that EGCG showed the highest MGO capture rate among flavonoids, followed by epicatechin, naringenin, kaempferol, trans-resveratrol, apigenin and fisetin, due to the flavanol structure of EGCG and the presence of a galloyl group at the C3 position of the heterocyclic C ring [63]. These indicate that the trapping of RCS by natural phenolic compounds is structurally dependent.

**Figure 3:**
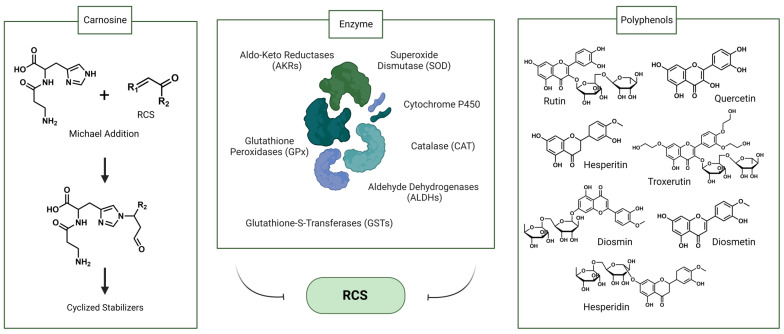
Key links affecting RCS metabolism can be divided into endogenous factors and exogenous factors. Endogenous factors mainly rely on carnosine and enzymes; during the exogenous metabolic process, dietary phenolic compounds are considered to be major RCS scavengers.

EGCG possesses multiple phenolic hydroxyl groups, which endow it with structural features enabling the trapping of RCS. Experiments investigating direct co-incubation of ACR and EGCG revealed a reduction in ACR by 71.60% within 1.5 h and 90.30% within 3 h, compared to the control [64]. This establishes EGCG’s potency as a natural scavenger, with the detailed process and mechanism of EGCG’s direct or indirect elimination of RCS to be further elucidated. (Figure 3). The potential correlation between EGCG intake and hepatotoxicity is noteworthy. Although the exact precise threshold for inducing hepatotoxicity remains undetermined, hepatic abnormalities were observed when daily intake exceeded or equaled 800 mg, and most cases were associated with green tea supplementation rather than consumption of green tea infusions [65].

## 4. Clearance Pathways of the RCS by EGCG

### 4.1. Source of EGCG

As a gallic catechin, EGCG is abundant in tea and berries, fruits, and nuts, including strawberries, blackberries, walnuts, and hazelnut kernels [66,67,68,69]. Research indicates that the presence of EGCG in non-tea foods is generally at modest levels, often falling below the 800 mg daily intake recommended by the European Union [70]. Moreover, Canada has implemented fresh guidelines for EGCG usage, capping the amount at 300 mg of EGCG and 600 mg of total catechin per day, along with 100 mg of epigallocatechin and 200 mg of total catechin per serving. Not only is the intake limited, but the bioavailability of EGCG in the human body through dietary sources is notably low. Based on the chemical characteristics, EGCG exerts its biochemical effects through direct binding and influence on signaling pathways. In antiviral and anti-inflammatory contexts, EGCG binds to the conserved cavity of the viral neuraminidase structure, effectively inhibiting the release of viral progeny from host cells [71]. EGCG’s binding to tumor necrosis factor receptor-associated factor TRAF6, especially to specific amino acids like Gln54, Asp57, and Ile72, significantly attenuates TRAF6’s E3 ubiquitin ligase activity, contributing to cancer prevention [72]. EGCG’s metal-chelating ability reduces metal toxicity in a non-selective manner. It can chelate trivalent iron ions, preventing conditions like Parkinson’s and Alzheimer’s by scavenging hydroxyl radicals [73].

Regarding regulatory approvals, in 2006, the European Parliament and Council enacted Regulation No. 1925/2006, permitting the addition of epigallocatechin gallate (EGCG) to food products. During food processing, EGCG can be added according to the legally stipulated content. In 2018, both EGCG and its lipophilic variant, such as EC16, were classified as non-toxic food additives. The United States Food and Drug Administration (FDA) recognized them as generally recognized as safe (GRAS) food additives, as indicated in FDA GRAS Notice 772. Furthermore, in 2019, the National Health Commission (NHC) of China, in collaboration with the State Administration for Market Regulation (SAMR), included EGCG in the sixth batch of the New Resource Food List. This inclusion explicitly allow for the addition of EGCG to food products in China. These regulatory approvals underscore the acknowledgment of EGCG’s safety and its recognized status as a permissible food additive in the European Union, the United States, and China. These regulations provide a regulatory framework for the controlled inclusion of EGCG in food processing, ensuring compliance with specified content limits.

### 4.2. Radical Capture

Based on the multiple hydroxyl groups and gallate moiety, EGCG can donate hydrogen atoms or electrons to scavenge free radicals, inhibit pro-oxidant enzymes, and activate antioxidant systems [74]. The B ring of EGCG serves as the major site of oxidation. The effective free radical scavenging activity of EGCG is ensured by the fact that the oxygen atom of the b-ring in EGCG interacts particularly strongly with the hydrogen atom of the gallic acid moiety [75,76]. The specific process involves initial single-electron oxidation of catechins at the B ring by 2,2-diphenyl-1-picrylhydrazyl (DPPH) radicals, yielding catechin phenoxy radicals. These phenoxy groups can undergo tautomerization, resulting in the corresponding o-quinone, and then subsequently engage in a nucleophilic attack on the quinone B ring through a Michael-type addition reaction, targeting the reactive C-8 (or C-6) carbon of another catechin unit [75,76]. However, observations from the H2O2 oxidizer system distinctly indicate the feasibility of oxidation on the A ring. Notably, EGCG’s presence can lead to the generation of endogenous H2O2, thus establishing its pro-oxidant behavior [77]. Reported investigations have demonstrated that pyrazine radical cations, as precursors to browning reactions, can be inhibited by EGCG [78]. These volatile compounds significantly impact the Maillard reaction and the formation of RCS. In a concentrated EGCG environment, phenols effectively quench the free radical precursors (glycolaldehyde, glyoxal) in a manner that binds to the highly reactive A-ring, thereby neutralizing their chemical reactivity. This essentially deactivates the conformation required for the production of pyrazine onium radicals, consequently effectively suppressing the formation of unsaturated aldehydes through free radicals, significantly impacting the Maillard reaction and the formation of RCS [78]. In food modeling studies, EGCG acted as an antioxidant, eliminating free radicals from the system to reduce the browning index of orange juice and pyrazine radicals in coffee [79]. Moreover, EGCG emerges as one of the most efficient singlet oxygen quenchers, with a practical application in physical quenching methods to diminish RCS production. For example, EGCG has demonstrated effectiveness in curtailing lipid peroxidation triggered by cigarette smoke extract and in reducing membrane peroxidation in NHBE cells. This attenuation effectively curbs the formation of 4-HNE.

### 4.3. RCS Inhibition in Maillard Reaction

Serving as a Maillard browning inhibitor, EGCG demonstrates the ability to interact with Amadori compounds and their subsequent degradation product, deoxypentose ketone [80]. EGCG primarily inhibits the formation of Amadori compounds through two pathways. Firstly, EGCG can modulate the activity and expression of enzymes involved in the metabolism of Amadori compounds and AGEs, such as aldose reductase, glyoxalase I, and amadoriase. Secondly, EGCG facilitates the degradation of Amadori compounds by catalyzing the cleavage of the C-N bond between the sugar and the amino group, thereby restoring the original protein or amino acid structure. This process effectively reverses glycation damage, ultimately enhancing the functionality of glycated proteins [81,82,83,84]. EGCG also can form covalent bonds with Amadori compounds, primarily within the A ring [82]. For instance, in the xylose–alanine model, EGCG, as a nucleophilic reagent, undergoes electrophilic aromatic substitution reactions at the C6 or C8 sites of the A-ring with Amadori rearrangement products (ARP) or deoxypentosone (DP) to form ARP-EGCG and ARP-DP-EGCG adducts [82]. In the black garlic model, black garlic immersed in EGCG was able to bind to the Maillard reaction intermediates such as HMF and 3-DG, reducing the level of active carbonyl compound precursors and further inhibiting the generation of active carbonyl compounds [85].

Furthermore, EGCG interacts with the substrates of the Maillard reaction to inhibit the progression of the reaction and the formation of RCS. In the case of amino acid substrates, EGCG can bind to the amino groups of proteins or amino acids, such as lysine and arginine, preventing them from reacting with sugars. Regarding sugar substrates, EGCG undergoes nucleophilic substitution with sugars and forms adducts [86]. In a glucose model, EGCG reacts with glucose through a nucleophilic substitution at high temperatures for a short time, followed by a dehydration cyclization, resulting in the formation of EGCG–glucose adducts that prevent further glycation. In the presence of amino acids, EGCG competes with them for the binding to the sugar fragments, and the competition is influenced by the temperature. At low temperatures, the binding of EGCG to the sugar fragments is predominant, while at high temperatures, the reaction of amino acids with the sugar fragments is faster [87].

Interestingly, in the Maillard reaction process, the combined use of EGCG with other reaction inhibitors exhibits a synergistic effect. Data indicate that the combination of EGCG with cysteine significantly diminishes the generation of Maillard products in juice systems [78].

### 4.4. Direct RCS Capture

The bio-activity of EGCG mainly arises from the presence of electron-rich conjugated systems and electron-donating phenol groups. EGCG possesses a robust nucleophilic character due to its characteristic m-phenol structure, allowing it to react with electron-deficient RCS and form adducts, thus serving as an efficient RCS scavenger. The major active site of EGCG is at positions six and eight of the A-ring, the gallate ring does not play an important role in the trapping of RCS. The slightly alkaline pH can increase the nucleophilicity at positions six and eight of the A-ring of EGCG [64,88,89]. EGCG can trap HNE by forming compounds distinguished by highly stable carbon–carbon single bonds at C6 and C8 of the A-ring. This addition of a hydroxyl group in the A-ring to the carbon–carbon double bond of the aldehyde effectively inhibits HNE from producing hemiacetals [90]. EGCG’s binding with MGO also takes place at either the C6 or C8 position of the A ring, resulting in the formation of single or double MGO adducts [91]. Under neutral or slightly alkaline conditions, the generation of both mono-MGO-EGCG and di-MGO-EGCG adducts is facilitated [88]. Acting as an electron-rich nucleophile, EGCG exhibits a propensity for nuclear attack at the electron-deficient aldehyde carbonyl carbon, especially at its C8 position [92]. Upon capture, MDA is prone to decompose into formic acid and acetaldehyde, or engage in reactions in forms such as monomers, dimers, and trimers, contributing to the formation of EGCG-MDA adducts [93].

EGCG tends to demonstrate heightened efficiency and a faster rate of removal towards low-molecular-weight RCS, including glycolaldehyde, acetaldehyde, acetone, and formaldehyde. It was discovered that a single gallic acyl group could effectively trap two molecules of ACR, while a single EGCG molecule has the potential to trap up to three molecules of ACR [94]. For the MGO, EGCG’s capturing ability of MGO surpasses that of amino acids [88]. EGCG can efficiently trap over 90% of MGO within 10 min at pH 7.4, 37 °C. In comparison to epicatechin (EC), epigallocatechin (EGC), theaflavins (TF), and theaflavin-3-gallate (TFG), EGCG exhibits the highest removal rate for ACR. Under conditions of pH 7.4 in PBS at 37 °C for 1.5 h, the removal rate surpasses 70%. Furthermore, EGCG’s removal rate for HNE at pH 7.4, 37 °C, over 24 h, is second only to TFG, at around 30% [95]. Furthermore, each hydroxyl group in the meso-site of phloroglucinol could eliminate one molecule of aldehyde, which suggests that one molecule of EGCG possesses the capability to eliminate octameric aldehydes [96]. The adduct formed by EGCG and small-molecule RCS can undergo further stabilization through hemiacetal and acetylation reactions subsequent to its formation. These suggest that EGCG, as an effective low-molecular-weight RCS scavenger, holds a distinct advantage. However, EGCG manifests reduced efficacy and a slower removal rate towards high-molecular-weight RCS, such as glycation end products [97]. For example, in the same model and environment, luteolin and rutin can individually inhibit AGE formation by 82.2% and 77.7%, respectively. However, under equivalent conditions, EGCG exhibits a lower inhibition rate of only 69.1% for AGEs [98].

## 5. EGCG-RCS Adducts Metabolism

### 5.1. Metabolites of EGCG Capture RCS

The small intestine serves as the primary site for EGCG absorption. In small intestinal epithelial cells, EGCG undergoes glycosylation and sulfation. Subsequently, EGCG is transported to the liver and then released into the bloodstream for distribution to other organs. Some metabolites are excreted via urine, while others exit the liver via bile, entering the intestine [99]. A significant portion of EGCG excreted into the ileum is reabsorbed for enterohepatic circulation. Unabsorbed EGCG traverses the colon and is eventually excreted in feces. The bioavailability of EGCG is known to be poor, with TPs absorbed through the intestinal wall at about 2–20% [100]. With the contribution of gut microbiota, it is reported that MGO-conjugated metabolites of EGCG were detected in urine and fecal samples collected from experimental subjects after consumption of commercial green tea, suggesting that RCS can be cleared by ingesting adequate amounts of EGCG [89]. Methods to improve the bioavailability of EGCG are food nanotechnology applications and structural modifications, including methylation and glycosylation [101].

EGCG undergoes metabolic reactions such as methylation, glucuronidation, and sulfation in animals, but the possibility of RCS capturing by EGCG in vivo that has undergone metabolism has been the subject of much research [102]. It has been observed that EGCG can be readily methylated by hepatic cytoplasmic catechol-O-methyltransferase (COMT) to form mono-4″-O-methyl-EGCG (MeEGCG), which subsequently transforms into 4′,4″-di-O-MeEGCG. In the excreta of the mouse model, ACR-4′-O-MeEGCG as well as mono-ACR-3′,4′-O-diMeEGCG can be detected [103]. This phenomenon is attributed to the fact that methylation of EGCG typically occurs at the B-ring or the D-ring, and the A ring of methylated EGCG in vivo retains its ability to capture RCS. In the anaerobic gut, EGCG can also react efficiently with ammonia to produce an aminated metabolite of EGCG (4′-NH 2 -EGCG) with RCS trapping ability by the A ring and primary amine group [93].

### 5.2. EGCG-RCS Adducts Metabolism

Various phenolic compounds exhibit distinct metabolic characteristics when forming adducts with RCS. Notably, recent studies showed that in the case of rutin–acrolein adducts, their absorption and metabolism in rats significantly differ from their precursor, rutin. This dissimilarity is evident in terms of absorption form, absorption speed, and in vivo retention. While rutin’s primary metabolites differ from those of the rutin–acrolein adducts, the latter are absorbed in their prototype form, and their absorption occurs at a much faster rate and exhibits prolonged retention within the body [104]. These findings suggest that the bioactivity of polyphenols may undergo substantial changes when they interact with RCS to form adducts.

**Figure 4:**
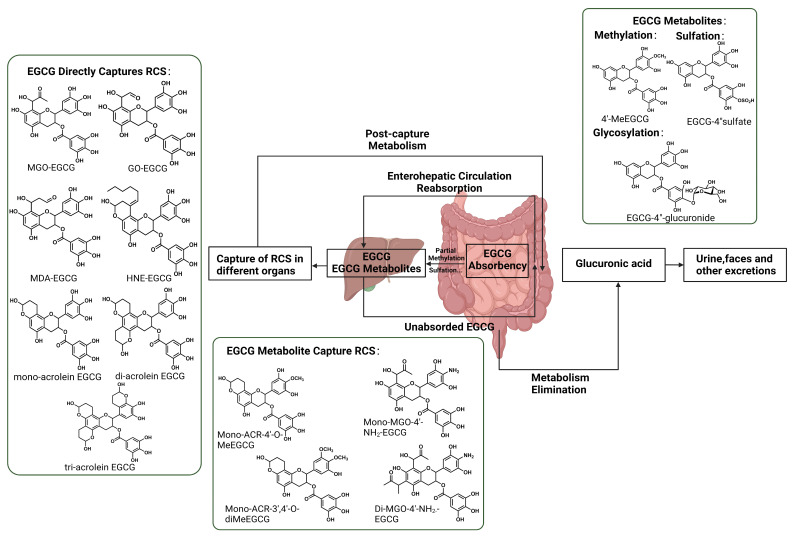
The main adduct of EGCG with MDA, MGO, ACR, and HNE, the main absorption metabolic pathway of the adduct in vivo.

Currently, the mechanism of EGCG capturing RCS has been increasingly elucidated. However, the metabolic process following the formation of these adducts within the human body remains obscure. Studies on mice suggest that EGCG-RCS adducts are excreted via urine or stool, facilitated by gut microbiota activity. Gut microbiota are able to secrete a variety of glycosidases, β-glucuronidases, sulfatases, and different esterases to hydrolyze EGCG, followed by cleavage of the C-ring, and finally further modification of the reaction products by dehydroxylation of the B-ring, and lactonization in the A-ring. EGCG is normally metabolized to phenyl-gamma-valerolactones, which are processed in the liver and excreted in the urine as glucuronidated and sulfated [105,106]. In the case of ACR, recent studies have revealed that EGCG reacts with ACR to generate ACR conjugates, ultimately excreted in urine as glucuronic acid. Within 24 h after EGCG administration, mono-ACR-EGCG was excreted as glucuronic acid in the urine of mice [103]. The urinary excretion of ACR-EGCG per time interval was as follows: 4.87 ± 2.5 nmol (ACR-Genistein) from 0–4 h, 3.14 ± 2.4 nmol (ACR-Genistein) from 4–8 h, and 19.9 ± 6.6 nmol (ACR-Genistein) from 8–24 h [103]. There is also a possibility that unabsorbed EGCG forms complexes with ACR in the gastrointestinal tract, eventually being excreted in feces. Unlike rutin–acrolein metabolism in rats, EGCG-ACR metabolism in rats produces EGCG metabolites and captures intestinal ACR [107]. While the gut microbiota’s role in producing dehydrogenistein (DGEN) and 6′-hydroxy-O-demethylangolensin (6′-OH-DMA) from genistein is recognized as an ACR elimination mechanism, the contribution of gut microbiota to the formation of EGCG conjugates with toxic active substances remains inadequately documented. Although data are limited, indications exist that the gut microbiota significantly participate in trapping toxic reactive substances using EGCG (Figure 4) [89].

## 6. Discussion

In recent years, with an increased awareness of the toxicity of RCS, numerous studies have focused on the efficacy and mechanisms of natural compounds in eliminating RCS. Among the compounds garnering widespread attention, EGCG demonstrates distinct advantages in RCS clearance compared to other natural phenolic compounds. Firstly, EGCG has been approved as a legal food additive in multiple countries, including the European Union, the United States, and China. Secondly, EGCG employs various pathways to inhibit RCS formation, such as suppressing precursor substance generation, disrupting formation conditions, and forming stable adducts. These pathways encompass scavenging free radicals and metal ions that induce RCS generation, inhibiting the substrates and intermediates of the Maillard reaction, and directly forming stable adducts with RCS. Furthermore, upon entering the human body, EGCG reduces blood glucose, thereby inhibiting the precursor generation of RCS. It activates the Nrf2/ARE signaling pathway and suppresses inflammatory reactions induced by RCS, collectively mitigating RCS-induced damage [108]. In terms of clearance efficiency, EGCG surpasses other phenolic compounds, exhibiting higher clearance efficiency, faster reaction rates, and greater affinity for RCS. This can be attributed to the presence of two hydroxyl groups in the A ring of EGCG, representing the primary active site for RCS capture. In addition to its comparative advantages over other phenolic compounds, EGCG exhibits enhanced reactivity in capturing MGO or GO compared to lysine and arginine. Furthermore, it demonstrates superior reactivity in capturing MGO compared to the pharmaceutical aminoguanidine, a compound known for inhibiting the formation of AGEs through the trapping of reactive dicarbonyl compounds in vivo [88,109]. It is noteworthy that EGCG exhibits robust RCS clearance activity even in alkaline environments. Some phenolic compounds tend to lose protons and form phenoxide ions, making their aromatic rings susceptible to nucleophilic attacks, and the substituents on the aromatic ring prone to reactions under alkaline conditions. These factors can impact the chemical properties of phenolic compounds, leading to a decrease in activity. However, EGCG demonstrates strong RCS clearance ability under alkaline conditions. This is attributed to the release of protons from the hydroxyl group at either C5 or C7 in the EGCG molecule under weak base conditions. This release prompts electron rearrangement and aggregation at C8 or C6, thereby promoting nucleophilic reactions [82]. It was reported that EGCG can effectively clear RCS, including MGO and GO, at pH 7.4, forming stable adducts. Additionally, EGCG demonstrates notable RCS clearance activity even at high temperatures. In the glucose model, EGCG engages in a nucleophilic substitution reaction with glucose at elevated temperatures for a brief duration. Subsequently, a dehydration cyclization occurs, leading to the formation of EGCG–glucose adducts that effectively inhibit further glycation processes. In the presence of amino acids, EGCG competes with them for binding to sugar fragments, and this competition is temperature-dependent. At lower temperatures, the binding of EGCG to sugar fragments predominates, while at higher temperatures, the reaction of amino acids with sugar fragments becomes faster.

The promising therapeutic potential of EGCG in mitigating the harmful effects of RCS raises important questions regarding its stability and safety within different food preparation settings. As previously discussed, researchers have noted discrepancies in the carbonyl phenol adducts formed in laboratory-fried onions versus industrially fried onions when quercetin is introduced. These variations, specifically in the type of aldehyde (2-enal versus acrolein) involved in the adduct formation, highlight the potential impact of food preparation methods on the composition of RCS adducts [110]. This phenomenon underscores the importance of considering food safety when incorporating EGCG into food products. Drawing parallels to the quercetin example, the inclusion of EGCG in food preparation could yield diverse EGCG-RCS adducts depending on the cooking conditions. This variability could raise concerns about the consistency and safety of food products enriched with EGCG. Once EGCG-RCS adducts form, the presence or absence of additional reactive groups becomes pivotal in assessing their stability. Furthermore, aldehydes derived from lipids in food products may undergo degradation under typical cooking conditions, contributing to the formation of novel carbonyl phenol mixtures. This phenomenon could further complicate the stability of EGCG-RCS adducts within the food system. In light of these considerations, the preservation of EGCG-RCS binding stability within food products emerges as a significant concern. It becomes crucial to explore methods to ensure the consistent formation and stability of these adducts in various culinary scenarios. Future research should investigate food processing methods that maximize the formation and stability of EGCG-RCS adducts, potentially involving variations in cooking temperatures, times, and other culinary parameters. Additionally, the development of sensitive and reliable analytical techniques for monitoring EGCG-RCS adducts in food products is essential.

In conclusion, existing research indicates that EGCG possesses distinctive features and advantages in inhibiting RCS compared to other naturally occurring phenolic compounds. It shows great promise in mitigating health issues related to RCS. Therefore, EGCG holds significant application value and market potential in food processing. Building on studies focusing on EGCG’s ability to clear RCS, it is essential to fully consider the combined effects of various phenolic compounds on RCS clearance and their underlying mechanisms. Furthermore, in terms of application safety, the European Commission introduced new regulations (EU) 2022/2340 in 2022, based on safety assessments by the European Food Safety Authority (EFSA), proposing that the daily intake of EGCG should not exceed 800 mg. This suggests potential risks associated with the use of EGCG. Therefore, when utilizing EGCG as a food additive, it is crucial to thoroughly consider the dose–response relationship and health risks associated with its consumption. Differing from traditional functional factors directly regulating physiological functions, the primary mechanism of RCS scavengers, such as EGCG, is the formation of stable adducts with RCS. Hence, research efforts should also consider the health risks associated with the formation of adducts between EGCG or other dietary phenolic compounds and RCS. It is imperative to clarify the biological activity or physiological function differences among different adducts, guiding the further development and application of RCS scavengers.

## Data Availability

The original contributions presented in the study are included in the article, further inquiries can be directed to the corresponding author.

## Abbreviations

1-DG	1-deoxyglucosone
ECGT	1-o-gallacyl-β-D-glucose o-galloyltransferase
ABTS	2,2′-azinobis(3-ethylbenzothiaziline-6-sulfonate);
DPPH	2,2-diphenyl-1-picrylhydrazyl
3-DG	3-deoxyglucosone
DGO	3-deoxyglucosone
4CL	4-coumarin-Coenzyme A
HHE	4-hydroxy-2-hexenal
HNE	4-hydroxy-2-nonenal
HMF	5-hydroxymethylfurfural
6′-OH-DMA	6′-hydroxy-O-demethylangolensin
APAO	acetylpolyamine oxidase
GA	acid-derived gallic acid
ACR-Gen	ACR adduct of genistein
ACR	acrolein; NAD-binding, adenine dinucleotide -binding
AGEs	advanced glycation end products
ALEs	advanced lipoxidation end products
ALDH1A1	aldehyde dehydrogenase 1 family member A1
ALDH1A3	aldehyde dehydrogenase 1 family member A3
ALDH4	aldehyde dehydrogenase 4
ALDHs	aldehyde dehydrogenases
AKR1B1	aldo-keto reductase family 1 member B1
AKR7A2	aldo-keto reductase family 7 member A2
AKRs	aldo-keto reductases
ARP	Amadori rearrangement products
AA	arachidonic acid
Arg	arginine
CO	carbon monoxide
CML	carboxymethyl lysine
CAT	catalase
C4H	cinnamate 4-hydroxylase
DGEN	dehydrogenistein
DP	deoxypentosone
DMS	dimethyl sulfide
EGCG	epigallocatechin-3-gallate
FA	formaldehyde
GLA	gamma-linolenic acid
GSH	glutathione
GPx	glutathione peroxidases
GSTs	glutathione-s-transferases
GS-DHN	glutathionyl-1,4-dihydroxynonane
GO	glyoxal
GOLD	glyoxal lysine dimer
H2S	hydrogen sulfide
LA	linoleic acid
LOOH	lipid hydroperoxides
Lys	lysine
MDA	malondialdehyde
MGO	methylglyoxal
MOLD	methylglyoxal lysine dimer
MFN2	mitofusin-2
MAPK	mitogen-activated protein kinase
MeEGCG	mono-4”-O-methyl-EGCG
NADPH	nicotinamide adenine dinucleotide phosphate
NHBE	normal human bronchial epithelial
NF-κB	nuclear factor κB
OPA1	optic atrophy-1
PAL	phenylalanine ammonia-lyase
Pro	proline
PKC	protein kinase C
RCS	reactive carbonyl species
ROS	reactive oxygen species
RAGE	receptor for advanced glycation end products
RCS-BSA	reduced, carboxamidomethylated, and succinylated bovine serum albumin; RyR2, ryanodine receptor 2
SSAO	Semicarbazide-sensitive amine oxidase
SSAT	spermidine/spermine N1-acetyltransferase
SMO	spermine oxidase
SOD	superoxide dismutase
Trx	thioredoxin
TrxR	thioredoxin reductase
Thr	threonine
MUC	trans-muconaldehyde
TRAF6	tumor necrosis factor receptor-associated factor 6
CsSCPL1A	type 1A serine carboxypeptidase-like acyltransferases
VAP-1	vascular adhesion protein 1
β-G	β-glucose gallate
